# Interactions of Short-Term and Chronic Treadmill Training With Aging of the Left Ventricle of the Heart

**DOI:** 10.1093/gerona/glv093

**Published:** 2015-08-05

**Authors:** Richard D. Walton, Sandra A. Jones, Kerry A. Rostron, Anna C. Kayani, Graeme L. Close, Anne McArdle, Matthew K. Lancaster

**Affiliations:** ^1^School of Biomedical Sciences, University of Leeds.; ^2^Department of Biological Sciences, University of Hull.; ^3^Institute of Ageing & Chronic Disease, Faculty of Health and Life Sciences, University of Liverpool.

**Keywords:** Aging, Exercise, Fibrosis, Calcium regulation, Hypertrophy

## Abstract

With aging, there is a decline in cardiac function accompanying increasing risk of arrhythmias. These effects are likely to be mechanistically associated with age-associated changes in calcium regulation within cardiac myocytes. Previous studies suggest that lifelong exercise can potentially reduce age-associated changes in the heart. Although exercise itself is associated with changes in cardiac function, little is known about the interactions of aging and exercise with respect to myocyte calcium regulation. To investigate this, adult (12 months) and old (24 months) C57/Bl6 mice were trained using moderate-intensity treadmill running. In response to 10 weeks’ training, comparable cardiac hypertrophic responses were observed, although aging independently associated with additional cardiac hypertrophy. Old animals also showed increased L- and T-type calcium channels, the sodium–calcium exchange, sarcoendoplasmic reticulum calcium ATPase, and collagen (by 50%, 92%, 66%, 88%, and 113% respectively). Short-term exercise training increased D-type and T-type calcium channels in old animals only, whereas an increase in sodium–calcium exchange was seen only in adult animals. Long-term (12 months) training generally opposed the effects of aging. Significant hypertrophy remained in long-term trained old animals, but levels of sarcoendoplasmic reticulum calcium ATPase, sodium–calcium exchange, and collagen were not significantly different from those found in the adult trained animals.

Dynamic exercise is considered to be a beneficial stress to the cardiovascular system. Current American College of Sports Medicine guidelines recommend adults undertake 30 minutes of moderate-intensity exercise most days of the week (1). Engaging in regular physical activity has been shown to improve overall health (2), improve cardiac muscle function (3), and enhance cellular mechanisms protective against cardiac insult (4). It is also considered that regular exercise throughout the life span is associated with decreased propensity for cardiovascular disease and improved functional capacity (5). Some studies have shown that age-related alterations of gene expression in the heart can be ameliorated by lifelong voluntary exercise, suggesting that exercise may preserve the heart in a “youthful phenotype” (6).

Exercise training is associated with increased cardiac mass (7), reduced resting heart rate (8), and improved ventricular diastolic function (9). Ventricular myocytes hypertrophy in response to dynamic exercise (10), and some studies also report changes in [Ca^2+^]_*i*_ regulation and contractility; however there is considerable variation in results, perhaps reflecting variation in species, training protocols, and genders used. Myocyte contractility following exercise training has been shown to increase (11), decrease (12), or remain unchanged (13). These changes are likely to be, at least in part, due to alterations in the underlying control of intracellular calcium; however, L-type Ca^2+^ channel protein expression has been reported not to change in response to exercise (14,15), but there is a mixed picture of possible changes to other processes controlling intracellular calcium. Sarcoendoplasmic reticulum calcium ATPase (SERCA2a) protein expression is reported to either increase (11) or remain unchanged (12,16); and sodium–calcium exchange (NCX) protein expression has been shown to decrease (12) or remain unchanged (17). One likely discrepancy between these results is the particular training protocols being used with studies, demonstrating wide variation in the training intensities, methods, and duration. Higher intensities of exercise appear to be associated with larger cardiac changes (18), but high-intensity training interventions do not reflect the current normal exercise recommendations or behaviors likely to be adhered to and indeed tolerated throughout the life span (1).

A further issue is that little is known about how adaptation of the heart to exercise changes with advancing age. Some studies suggest that the aged heart becomes resistant to adaptation and fails to respond to acute exercise stress like the young heart (19). However, the heart does show some maintained adaptability, and there is clear evidence of maintained cardioprotective effects from exercise even in old age, although perhaps as a result of a different balance of mechanisms from those induced in younger individuals (20).

The present study investigates responses to a moderate exercise intervention aimed to resemble a typical exercise recommendation (30 minutes of moderate-to-vigorous exercise at least three times per week). Adaptations to a moderate duration (10-week) exercise program in adult and old mice were assessed and compared with a long-term 12-month training intervention from adulthood to old age. The aim was to identify changes in the expression of proteins regulating intracellular calcium (and by inference cardiac muscle contraction and electrical stability of the heart) with aging and how these are modified by exercise training.

The hypothesis was that aging would result in changes with potentially negative implications for regulation of intracellular calcium. These in turn would be ameliorated by exercise training showing mechanistically how exercise can maintain functionality and stability in the aged heart.

## Methods

Adult (12–14 months) and old (24 months) male C57Bl6 mice (Charles River, UK) were housed singly in a specific pathogen-free environment with a 12-hour light/dark cycle. The study utilized an exercise protocol, which has been described previously (21). Mice were block randomized into five groups. The “trained” groups of mice consisted of adult mice subjected to a 10-week training protocol, old mice subjected to the same training protocol (timed such that the protocol finished as they reached 24–26 months of age), and adult mice subjected to a 12-month training protocol (to the age of 24–26 months). Two additional groups of adult and old mice were housed individually serving as age-matched untrained controls.

Training consisted of treadmill running on a motorized treadmill (Columbus Instruments, OH) with a 0% gradient at 15 m/minute for 15 minutes on 3 days/week. Mice were acclimated to the treadmill at speeds of 9–14 m/minute during the first 2 minutes of the 15-minute training session. Twenty-four hours after the final treadmill run, a cohort of mice from each of the five groups was killed by cervical dislocation, and the heart was removed. Tibia were dissected and measured using a micrometer screw gauge. Animal procedures were performed in accordance with the United Kingdom Animals (Scientific Procedures) Act 1986 and approved by the local ethics committees at the University of Liverpool and University of Leeds.

### Histological Examination of Cell Sizes

The ventricles were dissected, snap-frozen in cryo-media, sectioned at 12 µm, and placed on polylysine-coated slides. Tissue sections were labeled using rhodamine-conjugated wheat germ agglutinin (Dako, Denmark) to identify sarcolemmal membranes (22). Slides were examined by laser scanning confocal microscopy (LSM510 Meta, Zeiss, Germany). Transverse sections of cells were confirmed by “z-stack” imaging of sections labeled with wheat germ agglutinin and the cross-sectional area of each myocyte identified as the area within the bordering wheat germ agglutinin label. The widest part of the cellular cross-section was taken as the cell width.

### Analysis of Protein Expression

Tissue was processed as described previously (22). Samples (50 µg of total protein/lane) were separated by electrophoresis under reducing conditions by 10% sodium dodecyl sulfate–polyacrylamide gel electrophoresis, followed by transfer to nitrocellulose membrane. The membrane was probed by antibodies (see details in *Antibodies Used*) applied in the presence of SuperBlock (Thermo Scientific, MA) with 0.05% Tween-20. Bands were detected by SuperSignal, West Pico (Pierce, Cheshire, UK) and analyzed by ImageJ. Densitometric analysis of all labeled protein in each lane of the blot was performed, and equal protein loading was confirmed by comparison with desmin protein levels in each lane (Desmin was found not to vary significantly between groups (see Supplementary Figure) and has previously been used as a loading/normalization control in other rodent aging studies (23)). All protein expression values were normalized to average levels of the protein as identified in the adult sedentary mouse.

### Antibodies Used

Antibodies to Cav1.2 (α1C), Cav1.3(α1D), and Cav3.1(α1G) were from Alomone Labs (UK). Antibodies to SERCA2A and phospholamban (PLB) were obtained from Badrilla (UK) and to NCX were from SWANT (Switzerland). Antibodies to desmin and heat-shock protein 47 (HSP47) were from Dako (Denmark) and Thermo Fisher Scientific Inc. (MA), respectively. A goat anti-rabbit horse radish peroxidase–conjugated secondary antibody was used for Western blot detection of protein (Dako—used at 1 in 2,000 dilution). Primary antibodies were applied at a dilution of 1 in 1,000 apart from those to HSP47, SERCA2a, and NCX, which were applied at 1 in 500 dilution.

### Assessment of Collagen Deposition

The protocol for Sirius red staining of collagen fibers has been described previously (24). Briefly, five 12-µm cryosections from cross-sections of the ventricles distributed approximately evenly, such that they covered regions from the base to the apex of the left ventricle, were fixed in Bouin’s solution before staining in 0.1% Sirius red solution for 60 minutes at room temperature. Sections were washed in 0.01M HCl before dehydration and mounted using Di-N-Butylate Phthalate in Xylene (DPX; Sigma, UK). Images of sections, taken under polarized light, were analyzed to assess the percentage area of each image displaying Sirius red staining with background subtraction accomplished by a standard triangle method in ImageJ.

To assess hydroxyproline content, 10mg of ventricular tissue from each heart was subjected to hydrolysis in 6 N HCl for 3 hours prior to determination of oxidized hydroxyproline by a colorimetric method using 4-(Dimethylamino) benzaldehyde (25). All samples were tested in duplicate and converted to amounts in micrograms per milligram of heart tissue according to a standard curve of known hydroxyproline concentrations.

### Statistical Analysis

Data are expressed as mean ± *SEM*. Statistical differences were assessed by multivariate analysis of variance with Sidak post hoc comparisons. Pearson correlations were performed to assess correlation between variables. Significance was determined at *p* < .05. *n* = 7 animals for each group.

## Results

### Hypertrophic Adaptation to Aging and Exercise

Exercise and aging were both expected to associate with significant cardiac hypertrophy. The data in Table 1 show that although overall a significant difference in body weight was seen between old animals and young animals (*p* < .001), no significant differences were seen between individual control and exercise trained groups. A significant difference in heart weight was identified between old and adult mice (*p* < .001) as well as between each exercise group and the respective aged sedentary group of mice. In adult mice, 10 weeks of exercise increased heart mass by 10±2% (*p* < .001), and in old mice, a similar 9±3% (*p* < .001) increase was observed. The cardiac mass of mice trained for 12 months was not significantly different from that of the short-term trained old animals.

**Table 1.** Average Heart Weight and Body Weight Along With Heart Weight to Body Weight Ratio and Heart Weight to Tibia Length Ratio for the Different Groups Studied (*n* = 7 in each case)

	Adult	Adult	Old	Old	Old
		10 Wk.		10 Wk.	12 Mths.
Body weight (g)	34.2±0.5	34.5±0.4	37.7±0.9	35.5±1.4	37.3±1.2
Heart weight (mg)	284±3.8	312±4.5*	336±12.9^†^	366±16.2*^,†^	362±13.6*^,†^
Heart weight : Body weight (mg/g)	8.3±0.2	8.9±0.3	9.1±0.2	10.3±0.3^†,^*	9.7±0.3^†^
Heart weight : Tibia length (mg/mm)	15.7±0.5	16.7±0.4	18.4±0.8^†^	19.6±0.7^†^	19.7±0.7^†^

*Notes:* Adults were at 12 months of age with a group subjected to 10 weeks of treadmill training (Adult 10 Wk.). The old animals were at 24–26 months of age with a group subjected to 10 weeks of treadmill training (Old 10 Wk.), whereas a further group had been trained for 12 months (Old 12 Mths.).

*Indicate a significant difference (*p* < .05) to the sedentary control group at the same age.

^†^Indicates a significant difference compared with the adult control group.

When comparing heart weight to body weight ratios, a significant difference was not identified between adult and old sedentary mice (*p* = .172), and this similarly was not affected by exercise training in the adult mice (*p* = .211 for adult sedentary vs adults trained for 10 weeks). The heart weight to body weight ratio was, however, significantly greater in old mice that had been exercise trained for 10 weeks compared with both adult groups and the untrained old mice, exercise associating with an on average 10±3% increase in heart weight to body weight ratio in the old animals. When using an alternate scaling correctional element, the heart weight to tibia length ratio, the older animals proved to have a significantly increased ratio compared with adult animals (by 16±3%, *p* = .008); however, no significant effect of exercise on this ratio was observed.

**Figure 1.**
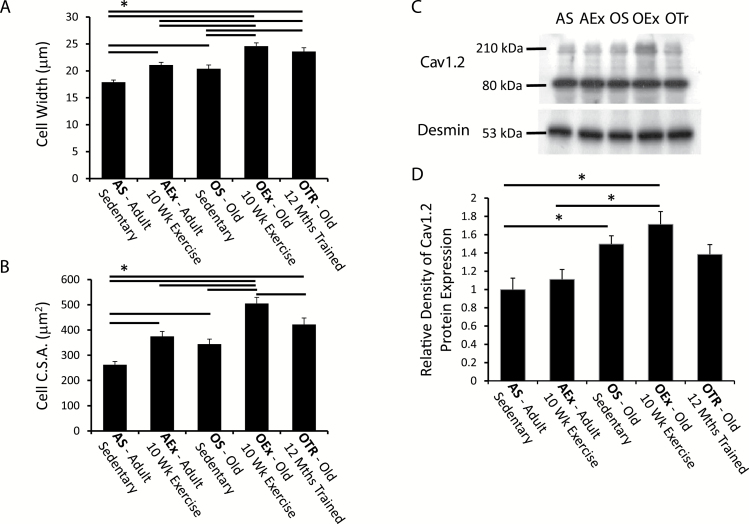
Myocyte cell width (**A**) and cross-sectional area (C.S.A.) (**B**) at each age and within each training group showing the exercise-induced hypertrophy alongside that associated with aging. The horizontal bars indicate significant differences between the groups (*p* < .05). (**C**) A typical Western blot for the L-type calcium channel alpha subunit Cav1.2 (upper image) alongside labeling for desmin (lower image). Samples from sedentary adult (AS), exercise trained adult (AEx), sedentary old (OS), exercise trained old (OEx), and chronically trained old (OTr) animals were run alongside one another on the same blots. (**D**) Average density of expression values normalized to the sedentary adult values.

Exercise, at the tissue level, was expected to lead to significant cardiac myocyte hypertrophy. Cross-sectional areas and cell widths of ventricular myocytes were found to significantly increase in response to both aging and exercise (Figure 1A and B). Ventricular myocytes from adult mice subject to 10 weeks’ exercise training had significantly greater transverse cross-sectional areas (42.8±7.5%, *p* < .001) and cell widths (17.9±2.9%, *p* < .001) compared with the ventricular myocytes from sedentary controls. This indicates the effectiveness of the moderate training program for stimulating cardiac adaptation. Old mice that had been sedentary, however, also had significantly greater myocyte cross-sectional areas and widths (by 31% and 14%, respectively) compared with the adult sedentary group, indicating a significant hypertrophic response associated simply with aging. Old mice that undertook the 10-week exercise program developed significant additional cellular hypertrophy. The magnitude of this response was similar to that seen in adult mice (cell width was 20.5% greater in the trained aged mice compared with sedentary aged mice) and appeared to simply be additive to the age-associated hypertrophy (Figure 1A), with the analysis indicating no significant interaction of exercise training and age. Significant hypertrophy was also evident in mice exercise trained for 12 months in which cell widths were significantly greater compared with adult and old sedentary mice. The myocyte hypertrophic response in mice exercised for 12 months was not significantly different from that observed in adult or old mice following 10 weeks of exercise in terms of cell width; however, cell cross-sectional area was significantly lower in the long-term trained mice compared with old mice trained for only 10 weeks.

The results confirm that aging and the moderate exercise stress used in this study are sufficient to induce physical adaptation of the ventricular myocytes but fail to indicate changes in exercise response with age or significant differences in hypertrophic adaptation to 12 months’ versus 10 weeks’ exercise.

### Expression of Ca_v_1.2—L-Type Calcium Channels

The L-type calcium current carried by Ca_v_1.2 is the primary controller of cardiac contractile response. Anti-Ca_v_1.2 immunolabeling of Western blots showed protein fragments at 210 and 80kDa as expected (Figure 1C). Denistometric analysis indicated that the training program had no significant effect on Ca_v_1.2 expression, a finding also previously observed by others (14). In contrast, aging associated with a significant increase in myocardial Ca_v_1.2 expression (Figure 1D). This increase was not significantly altered by 10 weeks of exercise training, although Ca_v_1.2 expression in mice trained for 12 months was not significantly different from that observed in adults.

### Expression of Ca_v_1.3—D-Type Calcium Channels

**Figure 2.**
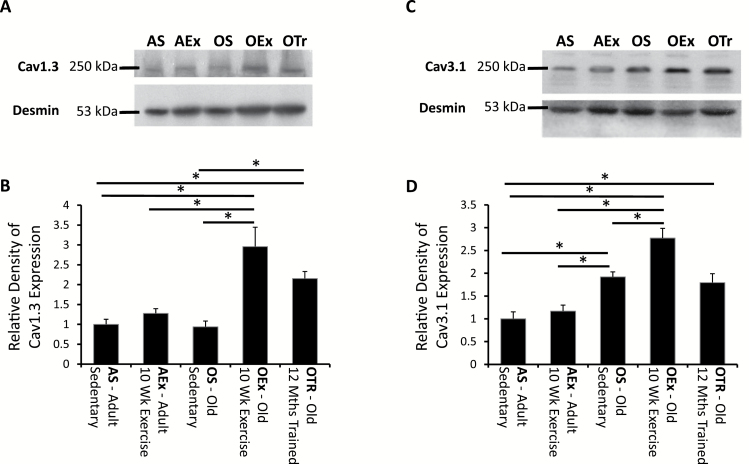
Representative blots showing expression of Cav1.3, the D-type calcium channel (**A**) and the Cav3.1 isoform of the T-type calcium channel (**C**). In each case, the same blot labeled for desmin is also shown. (**B** and **D**) The levels of each protein expressed relative to that found in the adult sedentary animal. The horizontal bars indicate significant differences between the different age and training groups (*p* < .05). AEx = exercise trained adult; AS = sedentary adult; OEx = exercise trained old; OS = sedentary old; OTr = chronically trained old.

Although Ca_v_1.2 is the predominant calcium channel in ventricular myocytes, other subtypes such as the D-type and T-type channels are present and have roles in signaling and modification of electrical activity (26,27). A single band at 250kDa was detected by the anti-Ca_v_1.3 antibody (Figure 2A). No changes in Ca_v_1.3 expression were observed with short-term exercise training in the adult mouse or with 12 months of sedentary aging (Figure 2B). In contrast, 10 weeks of exercise training significantly increased Ca_v_1.3 expression almost threefold in old mice. Similarly, mice that had been trained for 12 months showed greater cardiac expression of Ca_v_1.3 than either sedentary group or the adult mice that had been trained. This indicates a significant difference in adaptive response to exercise between adult and old individuals, whereby Ca_v_1.3 channel expression is induced only in the aged heart.

### Expression of Ca_v_3.1—T-Type Calcium Channel Subunit

Determination of Ca_v_3.1 revealed another pattern of calcium channel expression. Exercise in the adult mouse had no significant effect on Ca_v_3.1 expression, but sedentary aging almost doubled expression of this channel isoform (Figure 2D). Expression was increased significantly further by 10 weeks of exercise in the old mice; however, 12 months of exercise did not associate with an increase in expression beyond that observed simply with aging.

### Expression of NCX

**Figure 3.**
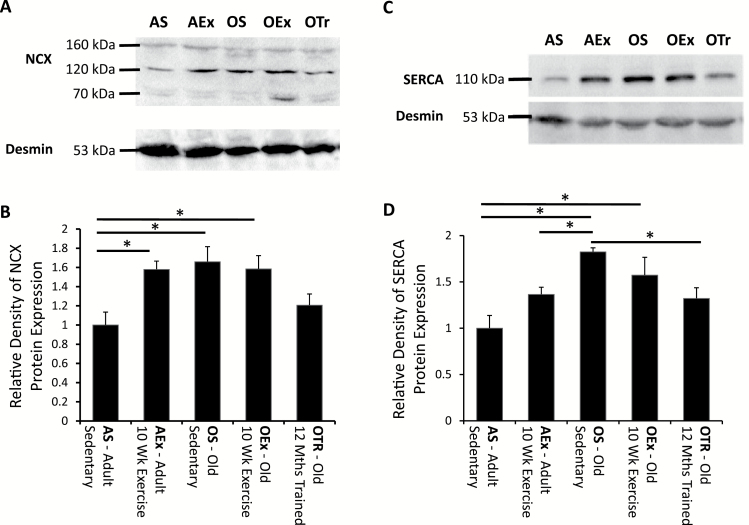
Exercise induced increase in NCX levels (**A** and **B**) and age-associated increased SERCA levels (**C** and **D**). (**A** and **C**) Typical blots for NCX (**A**) and SERCA (**C**) expression. The data are shown normalized to expression in the adult sedentary animals in **B** and **D**. The horizontal bars with asterisks indicate significant differences between groups (*p* < .05). AEx = exercise trained adult; AS = sedentary adult; OEx = exercise trained old; OS = sedentary old; OTr = chronically trained old.

NCX is the dominant sarcolemmal route for removal of cytoplasmic calcium in mouse cardiac myocytes. Western blotting identified 3–5 bands of the full protein, nonreduced protein, and proteolytic fragments, as observed by others (Figure 3A) (28). Ten weeks of exercise significantly increased expression of NCX in adult mice by 58±8.6% (Figure 3B). A similar increase was observed with aging, but exercise training of aged mice produced no further change in NCX expression. Twelve months of exercise training resulted in no difference in myocardial NCX expression compared with adult sedentary animals showing an apparent opposition to the normal aging response not observed with the short-term exercise intervention in old age.

### Expression of SERCA2a and PLB

Although NCX and the sarcolemmal calcium channels determine sarcolemmal balance of calcium fluxes, the principle source of calcium for myocyte contraction is the sarcoplasmic reticulum. SERCA2a expression was not significantly altered by 10 weeks of moderate exercise in adult mice; however, aging led to an increase in SERCA2a expression by 88±6.7% (Figure 3C and D). Expression levels were similar in old mice subjected to the 10-week training protocol, an intervention that had no apparent additional impact on SERCA2a expression. Longer-term exercise training, however, opposed the effect of aging on SERCA2a, and levels of SERCA2a in mice that had been trained for 12 months were not significantly different from those observed in adult mice.

**Figure 4.**
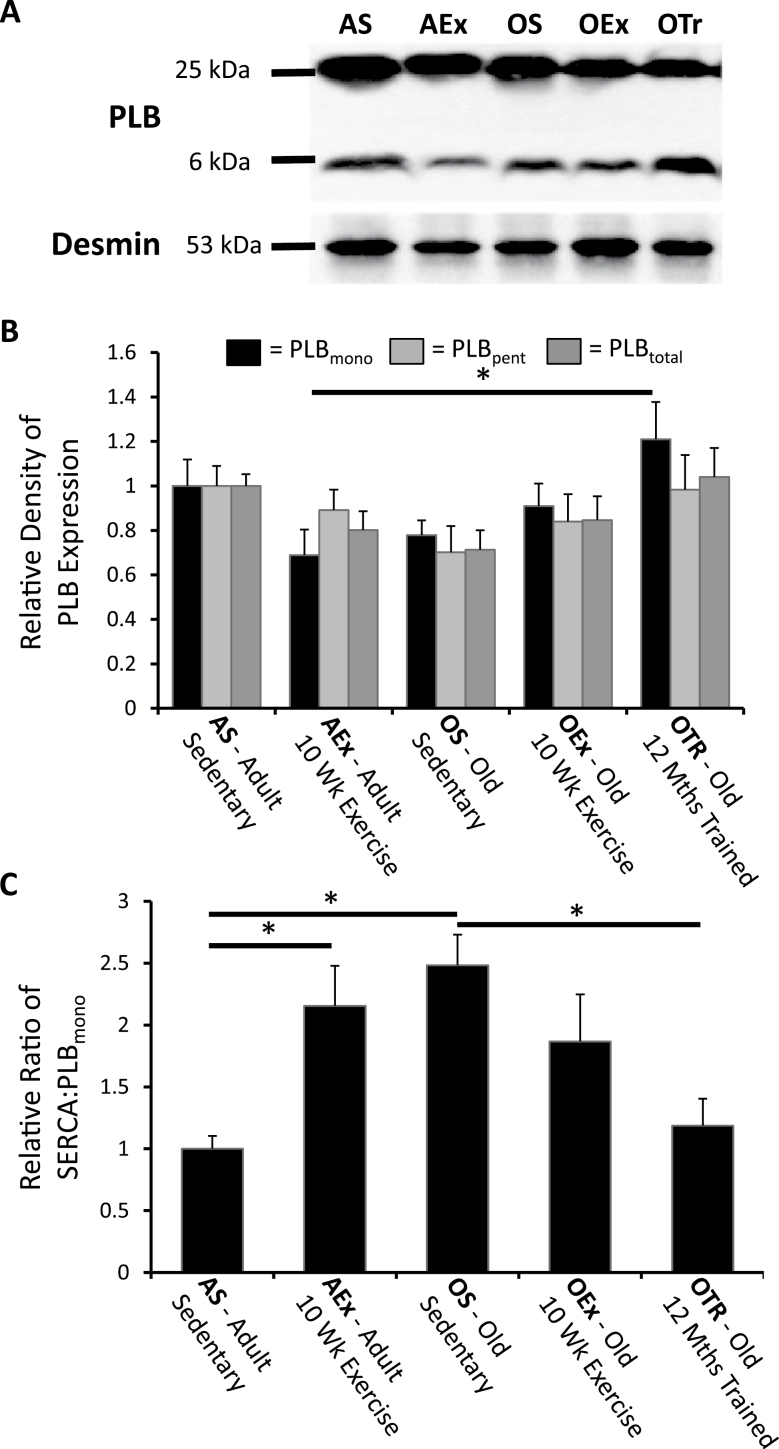
Expression of phospholamban (PLB) and the SERCA : PLB ratio. (**A**) A typical blot for PLB showing the separation of monomeric and pentameric forms, whereas (**B**) shows this data expressed relative to the adult sedentary control. Total PLB did not differ between any of the groups. (**C**) Shows that the ratio of SERCA to monomeric PLB is significantly elevated with aging and in adult animals in response to exercise training. In aged animals, chronic exercise opposed this change in ratio. Significant differences (*p* < .05) are indicated by the horizontal bars. AEx = exercise trained adult; AS = sedentary adult; OEx = exercise trained old; OS = sedentary old; OTr = chronically trained old.

Simple assessment of SERCA2a expression is not sufficient to indicate potential activity due to the normal background inhibition of SERCA2a by PLB. If PLB levels change with aging or exercise adaptation, this could adjust the overall expected activity of SERCA2a. Monomeric PLB binds to SERCA2a to cause inhibition but when not serving this role forms a pentameric molecule. Polyacrylamide gel electrophoresis under nonreducing conditions revealed both forms (Figure 4A), monomeric and pentameric, as well as total PLB expression did not change with age (Figure 4B).

### SERCA to PLB Ratio

The ability of PLB to regulate SERCA depends not only on phosphorylation status of PLB but also on the relative amounts of PLB to SERCA (29). The ratio of SERCA expression to PLB is displayed in Figure 4C. As expected, because there were no significant changes in PLB, the ratio approximates the observed changes in SERCA. The SERCA : PLB ratio was increased by 10 weeks of exercise in the adult mice, but there was no significant impact of short-term exercise in old mice. Aging alone was associated with a comparable significant increase in the SERCA : PLB ratio, but long-term exercise prevented this increase (Figure 4C).

### Age-Associated Fibrosis

**Figure 5.**
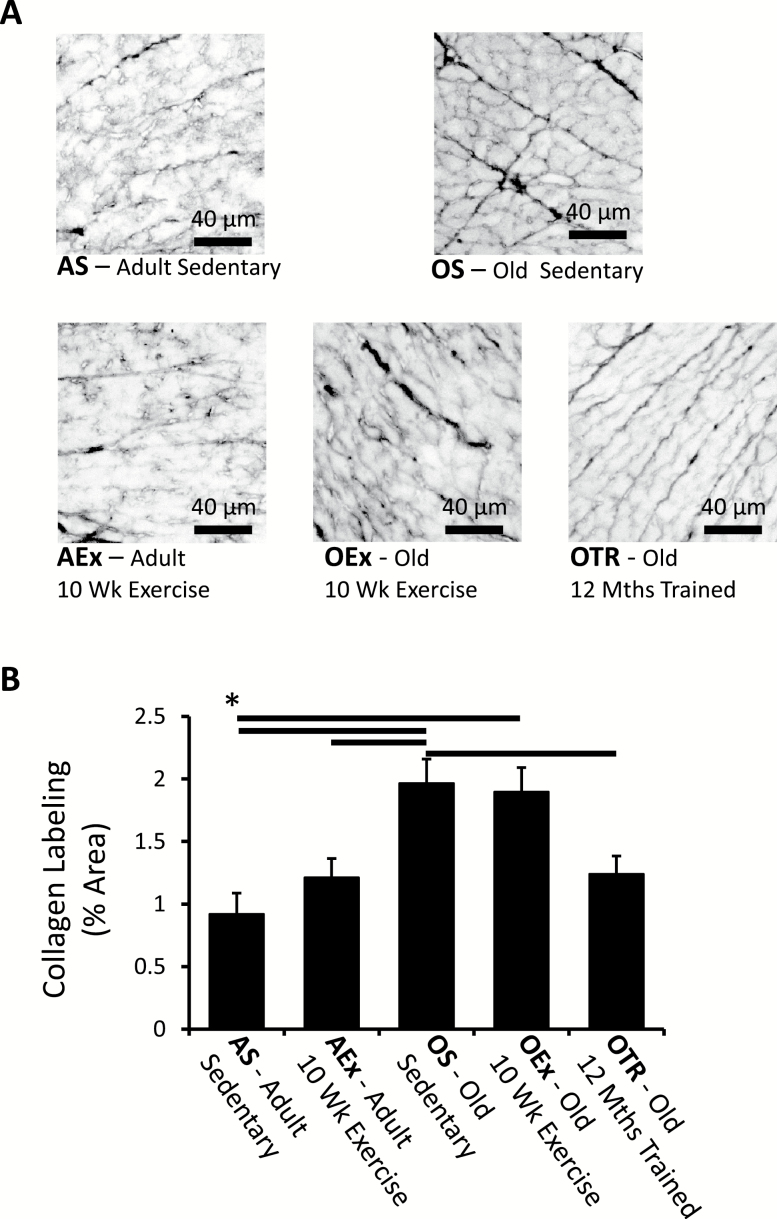
Typical images of picrosirius red labeling of collagen in sections from the left ventricle (**A**). (**B**) The percentage of the tissue area occupied by collagen normalized to that identified in sedentary adult animals. The horizontal bars show significant differences between groups. AEx = exercise trained adult; AS = sedentary adult; OEx = exercise trained old; OS = sedentary old; OTr = chronically trained old.

Previous reports have suggested that cardiac diastolic function and electrical conduction disruption may result from fibrosis of the ventricular tissue with aging (30). It, however, remains unclear whether cardiac fibrosis is an inevitable consequence of aging or secondary to other pathology. Example cross-sections of ventricles stained with sirius red are shown in Figure 5A. Aging associated with a significant increase in interstitial fibrosis; however, this was not significantly affected by short-term exercise (Figure 5A and B). Long-term exercise training, in contrast, reduced the appearance of age-associated interstitial fibrosis, and sections from these trained animals resembled labeling in the younger adults.

**Figure 6.**
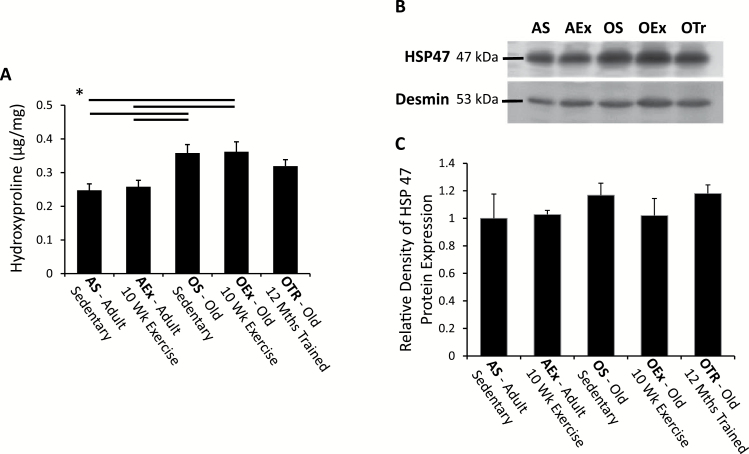
Markers of fibrosis in the heart. (**A**) Hydroxyproline content in micrograms per milligram of ventricular tissue. The horizontal bars show significant differences between groups. (**B**) A typical blot for HSP47 with quantified data in (**C**) shown normalized to the sedentary adult animal. AEx = exercise trained adult; AS = sedentary adult; OEx = exercise trained old; OS = sedentary old; OTr = chronically trained old.

To further quantify the apparent change in fibrosis, hydroxyproline (a major component of collagen) content of the tissue was assessed as shown in Figure 6A. Hydroxyproline was significantly elevated in old sedentary animals and old animals treadmill trained for 10 weeks compared with adult animals that were either sedentary or exercise trained, again indicating an age-associated accumulation of collagen. The 10-week training program had no significant effect on hydroxyproline content at either age studied. The 12-month training program, however, apparently ameliorated the fibrosis effect seen with age, and there was no significant difference in ventricular hydroxyproline content between this group and the sedentary adult animals.

To provide a further assessment of mechanisms associating with this fibrosis, we assessed expression of the collagen-specific chaperone HSP47, increasing expression of which is associated with fibrotic disease (31). Expression of HSP47 was not significantly different between all the groups examined (Figure 6B and C), possibly indicating a difference between the normal physiology seen here and fibrotic pathology.

## Discussion

An initial important conclusion from this data is that the aged heart remains responsive and adaptive to exercise showing comparable hypertrophic responses to short-term exercise stress with that identified in the younger animals. There are, however, also adaptations that appear to be age specific—those apparently induced by the exercise stress but not the aging process alone, and some that are additive. In particular, it is interesting to see that aging itself is associated with a similar degree of hypertrophy to that induced by the particular exercise regime utilized here, perhaps associating with the age-associated modest increase in body mass; however, exercise still creates a further additional effect that is superimposed on this. In both age groups, the training protocol had similar magnitude impacts on heart weight, directly observed at the cellular level as an impact on cell width.

### Calcium Channel Changes

Changes in L-type calcium current have been reported previously in cardiac myocytes with aging, although there is variation in the results with a general finding of a decline (eg (32)) but also studies reporting an increase (33) or no change (34). In the present study, an age-associated increase in expression of the alpha subunit of this channel was observed, which was not modified further by exercise. Such an increase in channel expression, if accompanied by enhanced calcium influx, may serve to help improve contractility, perhaps in the face of increased circulatory resistance, but apparently is not a recruited adaptive mechanism when faced with the overload of moderate exercise. Increasing calcium influx is an effective but potentially energetically expensive way to improve contractility, because in the steady state, influx must be balanced by extrusion of calcium ions from the cytoplasm with each heartbeat. As such, the age-associated increase could render the heart less energetically efficient. This inefficient mechanism of coping with changing cardiovascular demands is not recruited in normal adaptation to exercise.

Although the L-type calcium channel is the predominant calcium channel in the heart, D- and T-type channels have a role to play in the specialist conduction pathways of the heart, cell signaling, and as moderators of cardiac function (26,27,35). A novel finding is the induction of D-type calcium channel expression by exercise uniquely in the aged heart. D-type channels are closely related to the L-type channel but have altered sensitivity to calcium channel blockers and a lower activation voltage (36). Knockout of this channel has deleterious effects on pacemaking and electrical propagation, rendering animals susceptible to arrhythmias (27). The precise value of expressing higher levels of the D-type channel is difficult to ascertain, but we can speculate that it may be helping to ensure stability of the cardiac conduction system when the heart is under exercise stress. This may be particularly important for the aged heart, which we know is already at a higher risk of arrhythmias (37).

In a contrasting manner, the Ca_v_3.1 T-type channel subunit and associated T-type current may have a role in triggering adaptation and associated signaling cascades. Expression of this increased with aging, with short-term exercise stress (but not chronic training) creating a further induction of expression. Blockers of T-type current have been shown to be beneficial in heart failure and pathological adaptation, implying a negative role for the chronic induction of this channel (38). In the short term, however, the induction of T-type channels may simply have a role in instigating normal hypertrophic responses. The failure of chronic exercise to reverse the age-associated chronic induction of the T-type channel perhaps shows limitations in its ability to protect the heart against age-associated remodeling.

### Adaptations to Preserve Calcium Homeostasis

The aged heart shows signs of adaptation to cope with enhanced calcium fluxes. Enhanced NCX and SERCA expression in old age imply an increased ability to handle calcium ions, although this could potentially be at the cost of overall stability if calcium balance is disrupted. As a parallel to this, increased NCX expression is also a feature of end-stage heart failure and has been associated with increased risk of arrhythmias, an increased risk also seen in those who are old (39). A further problem affecting elderly individuals is a decreased response to adrenergic stimuli (40). The altered ratio of PLB expression to SERCA suggests that the potential control of SERCA is affected by aging potentially limiting the dynamic range of response, although such a change was also seen in the trained adult.

Overall, a key finding was the apparent ability of long-term exercise to reverse or prevent age-associated changes in these control proteins. Long-term exercise associated with NCX and SERCA expression levels not significantly different from those found in the sedentary adult as well as a comparable SERCA : PLB ratio. This suggests that long-term training has maintained a “youthful phenotype” with respect to the expression of key proteins regulating intracellular calcium balance potentially preserving stability and dynamic response. A similar outcome, however, cannot be recreated by short-term training.

### Exercise as a Modifier of Ventricular Fibrosis

The benefits of long-term training are not just seen with regard to apparent maintenance of expression levels of proteins responsible for calcium regulatory function. Systolic and diastolic function of the heart as well as electrical stability are affected by calcium regulation, but physical changes such as fibrosis also may play a role in establishing the aged heart phenotype (41). Interstitial fibrosis and collagen accumulation within the left ventricle during aging were prevented by long-term exercise, although not modified by short-term exercise. Our data show a potential mechanistic means whereby regular exercise can help maintain diastolic function in old age as well as protect against arrhythmias by reducing or preventing fibrosis. Although a limitation of the present study is a lack of functional data regarding diastolic function for this animal cohort, previous work has shown voluntary wheel running to associate with improved diastolic function in old age as shown in the same strain of mice, this despite an age-related decline in running activity (42).

Interestingly in the present study, the remodeling of collagen did not associate with changes in HSP47, which has been suggested to be a key pro-fibrotic signal to the point where suggestions have been made to reduce fibrosis therapeutically by targeting HSP47 (43). The present data, however, show that such therapies may be ineffective and that long-term exercise is apparently working via another unidentified mechanism to suppress the observed fibrosis.

A limitation to the current study is the lack of functional measures of calcium regulation and electrical stability with relation to the impact of training to confirm the impact of our observed changes. We know stroke volume is maintained in old age (44), and sarcoplasmic reticulum calcium content has previously been shown to be unaltered in aged mice (45), but the ventricle of aged mice remains more susceptible to arrhythmias when compared with adult mice (46), and this implies potential dysregulation of cellular calcium regulation. Further experiments are required to ascertain whether this is due to the changes identified here in expression of calcium-regulating proteins or whether other factors such as potassium-channel changes are key, such as has been proposed to differentiate pathological versus physiological hypertrophy (47). The use of a physiological mediator of cardiac hypertrophy, exercise, however, continues to offer a potentially valuable model to differentially investigate this and differentiate the underlying signals that may underlie the age-associated changes predisposing to pathology versus harmless or even beneficial physiological hypertrophy processes.

## Conclusions

Overall, the data show potentially deleterious changes in the heart with aging, many of which are directly opposed by long-term moderate exercise. The fact that the protocol was not progressive implies that mild sustained physical activity can be therapeutically beneficial. This coupled with the failure of short-term exercise training to produce the same benefits once the animal is already aged underlines the added benefits of regular exercise throughout the life span.

## Supplementary Material

Please visit the article online at http://gerontologist.oxfordjournals.org/ to view supplementary material.

## Funding

This work was supported by the British Heart Foundation (PG/11/15/28775), Wellcome Trust (WT086998MA), and SPARC.

## Supplementary Material

Supplementary Data
