# Supplementary material for: Interactions of Short-Term and Chronic Treadmill Training With Aging of the Left Ventricle of the Heart
Source: J Gerontol A Biol Sci Med Sci. 2015 Aug 5;71(8):1005–13. doi: 10.1093/gerona/glv093 (PMC4945880; doi:10.1093/gerona/glv093)
Supplement: Supplementary Data [file supp_71_8_1005__index.html]

Interactions of Short-Term and Chronic Treadmill Training With Aging of the Left Ventricle of the Heart — Interactions of Short-Term and Chronic Treadmill Training With Aging of the Left Ventricle of the Heart — Supplementary Data 

# Interactions of Short-Term and Chronic Treadmill Training With Aging of the Left Ventricle of the Heart

## Supplementary Data

Data files

- Supplementary Data - Supplementary Data
