# Supplementary material for: Interactions of Short-Term and Chronic Treadmill Training With Aging of the Left Ventricle of the Heart
Source: J Gerontol A Biol Sci Med Sci. 2015 Aug 5;71(8):1005–13. doi: 10.1093/gerona/glv093 (PMC4945880; doi:10.1093/gerona/glv093)
Supplement: Supplementary Data [file supp_glv093_Desmin_Control_Data.docx]

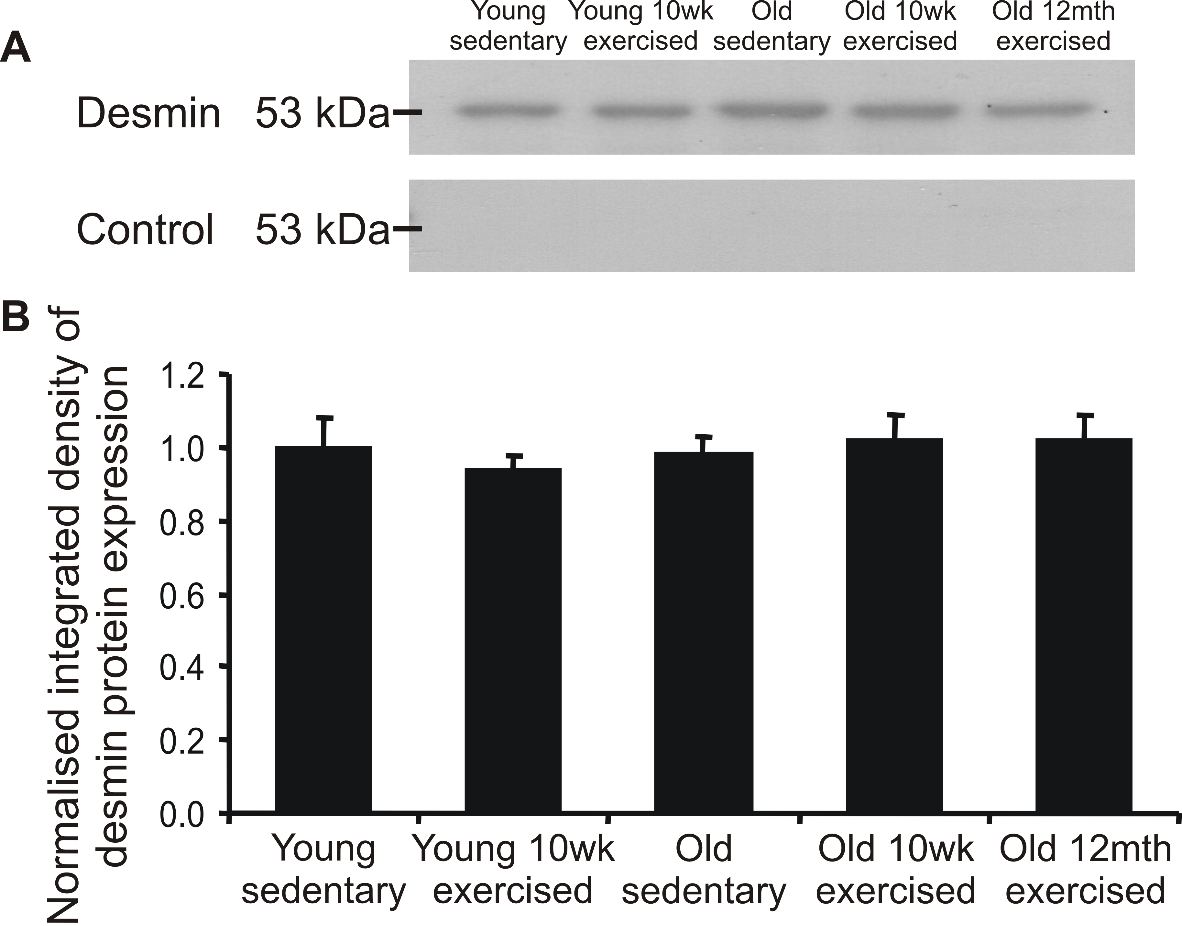


Desmin protein expression in the ventricles of young and old mice and following exercise.

(A) Western blots showing immuno-labelling of desmin from protein homogenates extracted from mouse ventricles. Desmin primary antibodies were omitted for controls. (B) The quantity of protein expressed in samples was measured as the integrated density of specific bands for desmin as detected by enhanced chemiluminescence. N of each group = 7. Data are means ± SD. No statistical difference was found by analysis of variance.
